# Factors Associated with the Choice of Peritoneal Dialysis in Patients with End-Stage Renal Disease

**DOI:** 10.1155/2016/5314719

**Published:** 2016-03-06

**Authors:** Pei-Chun Chiang, Jia-Jeng Hou, Ing-Ching Jong, Peir-Haur Hung, Chih-Yen Hsiao, Tsung-Liang Ma, Yueh-Han Hsu

**Affiliations:** ^1^Division of Nephrology, Department of Internal Medicine, Ditmanson Medical Foundation Chia-Yi Christian Hospital, 539 Zhongxiao Road, Chiayi City 60002, Taiwan; ^2^Department of Business Administration, National Chiayi University, Chiayi City, Taiwan; ^3^Department of Applied Life Science and Health, Chia-Nan University of Pharmacy and Science, Tainan, Taiwan; ^4^Department of Nursing, Min-Hwei College of Health Care Management, Tainan City, Taiwan; ^5^Department of Public Health and Department of Health Services Administration, China Medical University, Taichung City, Taiwan

## Abstract

*Background*. The purpose of this study was to analyze the factors associated with receiving peritoneal dialysis (PD) in patients with incident end-stage renal disease (ESRD) in a hospital in Southern Taiwan.* Methods*. The study included all consecutive patients with incident ESRD who participated in a multidisciplinary predialysis education (MPE) program and started their first dialysis therapy between January 1, 2008, and June 30, 2013, in the study hospital. We provided small group teaching sessions to advanced CKD patients and their family to enhance understanding of various dialysis modalities. Multivariate logistic regression models were used to analyze the association of patient characteristics with the chosen dialysis modality.* Results*. Of the 656 patients, 524 (80%) chose hemodialysis and 132 chose PD. Our data showed that young age, high education level, and high scores of activities of daily living (ADLs) were positively associated with PD treatment. Patients who received small group teaching sessions had higher percentages of PD treatment (30.5% versus 19.5%; *P* = 0.108) and preparedness for dialysis (61.1% versus 46.6%; *P* = 0.090).* Conclusion*. Young age, high education level, and high ADL score were positively associated with choosing PD. Early creation of vascular access may be a barrier for PD.

## 1. Introduction

The prevalence of end-stage renal disease (ESRD) is increasing steadily globally, with the highest one in Taiwan [1]. In the National Health Insurance (NHI) program in Taiwan, the costs of dialysis (both hemodialysis (HD) and peritoneal dialysis (PD)) are reimbursed by the government; therefore, the health care costs for ESRD patients are a heavy burden on society.

Compared with HD, PD is associated with the advantages of higher quality of life, preserved residual renal function, and cost saving [2]. Several studies reported a potential survival advantage over HD [2, 3]. However, currently the proportion of patients choosing PD is much lower than that of HD in Taiwan, around 16.4% in 2014 [4]. Previous studies have identified several crucial factors associated with renal replacement modality (RRM) selection, including the timing of referral, physician bias, predialysis education, resource availability, social and cultural habits, access to hospital dialysis beds, and lack of teamwork experience with certain dialysis modalities [5–7]. Barriers to PD include medical and social factors, physician bias, late referral, unplanned dialysis, time spent on modality education, and education not tailored to the needs of older patients [8, 9]. In addition, ESRD patients who had better family/caregiver support, higher cognition of dialysis, and stronger receptivity to dialysis were associated to the high percentage of PD treatment [10].

Before reaching the stage of dialysis-dependent ESRD, patients with chronic kidney disease (CKD) who receive early nephrology referral and multidisciplinary predialysis education (MPE) are associated with improved survival [11, 12], cost savings [5, 13, 14], and a significantly increased PD selection rate [5, 15, 16]. Previous studies have shown that, when offered a choice, approximately half of patients with incident ESRD choose home-based therapies, including PD [9, 17]. Hence, it is now widely accepted that MPE is an integral part of care for CKD patients before becoming ESRD and might improve PD uptake.

Studies from the United States [18] and Canada [19] have demonstrated that patients might favor one dialysis modality after predialysis education but ultimately choose another modality. We think that CKD patients might change the cognition of dialysis and receptivity to dialysis after MPE program and that might influence their choice of RRM. Therefore, we performed a retrospective chart review to identify the factors influencing ultimate dialysis modality for those who had received MPE prior to initiating regular dialysis. In addition, we had created a new MPE model, the small group session, to provide sufficient information about the cognition of dialysis to CKD stage 5 patient and estimate the efficacy of RRM selection.

## 2. Material and Methods

We retrospectively reviewed the demographic characteristics, personal disease loads, and final dialysis modalities of all patients who received the MPE program and started their first dialysis therapy in the study hospital between January 1, 2008, and June 30, 2013. The options of RRT in this hospital were renal transplantation, PD, and in-center HD, but not home HD. All patients received a regular health education program in their outpatient visits to assist their RRT modality choice. Final modality choice was made by the patients. All of the medical staffs were salaried employees. All nephrologists had been in practice for more than 6 years at the hospital and had practiced both PD and HD.

### 2.1. Participants

All study participants met the following criteria: (1) aged between 20 and 80 years; (2) received the MPE program and medical care more than 3 months before starting regular dialysis. The exclusion criteria were as follows: (1) patients transferred from other centers after starting RRT; (2) restarting dialysis after failure of a renal graft; (3) acute kidney injury with emergent dialysis; (4) loss to follow-up or mortality within 3 months of dialysis.

### 2.2. Multidisciplinary Predialysis Education

The education team comprised 5 nephrologists, 3 trained nurse educators (specialized in CKD management), 1 dietician, and 1 social worker. All CKD stages 3B to stage 5 patients received clinical evaluation and laboratory examinations and completed nursing and dietary education at least every 3 months. At every education session, the formal education and specialized topics targeted for individual patient conditions were delivered by one of the 3 trained nurse educators with a teaching time of 20–30 minutes. The contents of the education program included principles of dietary control, lifestyle modification, risk factors associated with renal function progression, pharmacological regimens, and avoiding nephrotoxic agents, according to relevant professional guidelines and Taiwan's pre-ESRD care program. Diabetes educators were also available for patients with diabetes. Patients with advanced CKD (CKD stage 5, or preuremic status) were followed up more frequently, on a monthly basis, and the MPE sessions focused on monitoring uremic complications, introducing RRM (HD, PD, and renal transplantation), early referral for dialysis access surgery, dialysis-associated complications, and the timing of initiating dialysis therapy. This education program ended once the patients started regular dialysis.

Small group teaching sessions were also available by January 1, 2011. All patients with advanced CKD, their family members, and caregivers were invited to the session. In the sessions, nephrologists provided professional recommendations and volunteer patients who had started HD or PD shared their experiences. We also arranged on-site visits to both PD and HD units. In the PD unit, PD nurse explained the facility of medical service (the teaching program of PD treatment, the delivery of dianeal, home visit, and the method of call for help) and PD modality (continuous ambulatory PD, automated PD, but not assisted PD) and provided the media (written and video in different languages) about PD treatment to patient and family/caregiver. The program lasted for 4 hours.

### 2.3. Definition and Data Collection

#### 2.3.1. Peritoneal Dialysis Patients

Criteria were patients who chose peritoneal dialysis for his/her long term dialysis modality and received regular dialysis for at least 3 months.

#### 2.3.2. Hemodialysis Patients

Criteria were patients who chose hemodialysis for long term dialysis modality and received regular dialysis for at least 3 months.

### 2.4. Traveling Time

We evaluated geographic factors using driving time but not distance with the Geographic Information System (GIS) technique. GIS has been shown to be an efficient method to determine the associations among geospatial distribution, socioeconomic, and health data [20]. The use of GIS in the field of public health has been constantly increasing in recent years [21, 22]. Various address georeferencing (AG) tools are now available, and Google Earth has been proven to be a good soft-web between AG tools [23]. Traveling time was the time data collected using commercial software (Google Earth) to map the location of patient address (Global Positioning System coordinates) and navigate to the hospital.

### 2.5. Activities of Daily Living (ADL)

The Barthel Index (BI) score system has been used to evaluate the ability of ADL in patients with stroke since 1965 [24]. The reliability and validity have been verified in various reports [25, 26]. The BI has also been adopted to evaluate the level of ADL for patients with CKD [27, 28]. BI is composed of 10 categories: 2 items are evaluated using a 2-point scale (0 and 5 points): grooming and bathing; 6 items are assessed using a 3-point scale (0, 5, and 10 points): bowel control, bladder control, dressing, feeding, toilet use, and climbing stairs; and 2 items are scored on a 4-point scale (0, 5, 10, and 15 points): moving from a wheelchair to bed and returning and walking on a level surface. The items are given a score for each category according to patient independence level. The range of the overall score is from 0 to 100, with a higher score indicating independent functional status in ADLs. All incident dialysis patients were assessed and scored using the BI system by trained nurses and were classified into 3 categories: independent ADL (BI score: 100), partial dependent ADL (BI score: 55–95), and dependent ADL (BI score: 0–50) [27].

### 2.6. Comorbid Disease

We defined comorbid disease by chart review. The diseases included hypertension, diabetes mellitus, coronary heart disease, congestive heart disease, and cerebral vascular accident.

### 2.7. Physician's Follow-Up

The in-charge nephrologist was the doctor responsible for the patients' outpatient services (OPS) in the 3 months before starting dialysis or who saw the patients in over 50% of the patients' OPS.

### 2.8. Dialysis Access Preestablishment

The patients underwent an operation for dialysis access before incident dialysis and had not used any temporary dialysis access.

### 2.9. Statistical Analysis

Means and standard deviations for continuous data and frequencies and percentages for categorical data are presented to demonstrate patient characteristics. Univariate analyses, including 2-sample *t*-tests for continuous variables and univariate logistic regression analysis or chi-square tests for categorical variables, were conducted to study correlations between factors and dialysis modality. Factors with significant correlations in univariate analyses were then included in the multiple logistic regression models to reverify the significance without a model selection process. Statistical analysis was conducted using SPSS version 12.0 (SPSS Inc., Chicago, IL, USA) and a 2-sided test with *P* < 0.05 was set as the cut-off value for statistical significance.

## 3. Result

**Figure 1:**
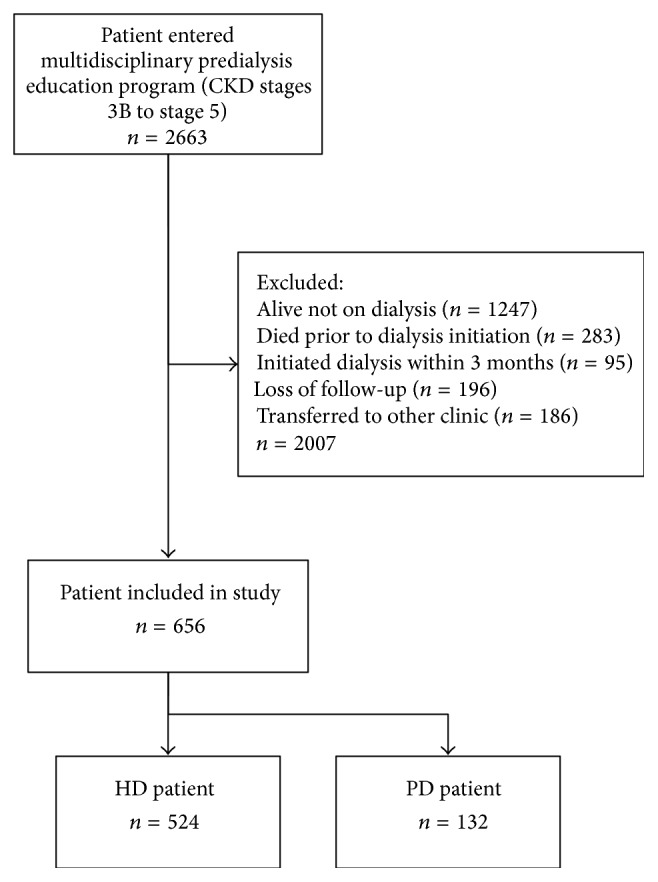
Study population selection.

In all, 656 of the 2663 incident dialysis-dependent ESRD patients who received MPE met the inclusion criteria and were enrolled for analysis. Reasons for exclusion are shown in Figure 1. Of the 656 enrolled ESRD patients, followed up by 5 different nephrologists, 524 (80%) chose HD and 132 (20%) chose PD.

For the PD and HD patient groups, respectively, the average age was 58.6 and 66.3 years, 55.3% and 54.8% were males, and most patients were married (87.9% and 89.7%), retired, or unemployed (71.2% and 85.7%) and had a lower level of education (including no education and primary school) (72.0% and 89.0%). Most of the ESRD patients in the 2 groups were complicated with comorbid diseases (85.6% in the PD and 88.0% in the HD group). Results of univariate analysis showed that age (*P* < 0.001), employment status (*P* < 0.001), educational level (*P* = 0.005), ADL status (*P* = 0.011), and dialysis access preparation (*P* < 0.001) were significantly correlated with dialysis modality selection. But physician bias (*P* = 0.256) and traveling time (*P* = 0.703) showed no significant differences (Table 1).

Young age, high educational level, and independent ADL were associated with PD treatment. Dialysis access preparation is a significant negative association factor with choosing PD (OR 0.350, 95% CI 0.226–0.542; *P* < 0.001) (Table 2).

Patients who received small group teaching sessions, compared with those who received only MPE, had higher percentages of PD treatment (30.5% versus 19.5%; *P* = 0.108) and preparedness for dialysis (61.1% versus 46.6%; *P* = 0.090), but the differences were not significant (Table 3).

## 4. Discussion

The initial choice occasionally differed from the choice of final treatment; therefore, we used the data from incident dialysis-dependent patients with ESRD to identify the factors influencing their RRT. We found that young age, high education level, and high ADL score were positively associated with choosing PD. Early creation of vascular access may be a barrier for PD.

Traveling time was not a critical factor influencing RRM selection in this study. Previous studies have shown that ESRD patients might focus on the distance to a dialysis center, and a modality fitting with lifestyle and distance to the dialysis center are crucial barriers to PD [29]. In a cross-sectional study in Central Taiwan, Huang et al. showed that short traveling time to a dialysis center was significantly correlated with PD treatment [10]. In Taiwan, many HD centers provide free transportation which may be an incentive for choosing HD. Hence, many HD patients (but not PD patients) with disability or who lived far away from the hospitals chose to receive HD at nearby dialysis centers after their conditions became stable. Therefore, the characteristics of ESRD patients who received regular HD in the hospitals might not be representative. In this study, we used the GIS data of incident patients to estimate the traveling time. The analysis results showed that traveling times were not significantly correlated with choosing PD. This finding was similar to findings in another recent study [19].

The proportion of PD in this study was 20%, higher than the national average of 16.4%. On further analysis, the PD proportion in all 5 nephrologists looked similar (*P* = 0.256). In previous surveys of nephrologists' attitude, patient preference and quality of life were the 2 major factors for RRM selection [30–32]; the least crucial factors were the dialysis cost and physician payment or facility reimbursement [30–33].

According to the guidelines of pre-ESRD care program in Taiwan, education on dialysis modality was arranged to stage 5 CKD patients only. Most CKD patients disliked doctors to mention the information about dialysis; therefore most patients initiated dialysis at a relatively delayed status. Hwang et al. reported that the nation-wide average eGFR for ESRD patients to start dialysis was 4.7 mL/min/1.73 m^2^ [34].

CKD patients who had predialysis education were more likely to choose the dialysis modality based on past experience of those who were already on dialysis or the opinions from their family or friends, rather than the opinion of the medical staff [35, 36]. Likewise, in our study, physician recommendation was not a major factor influencing the treatment of RRM.

Patients with independent ADL were associated with a higher percentage of PD therapy than those with partial dependent ADL. A systematic review of qualitative study showed that CKD patients and caregivers recognize that home hemodialysis might provide the opportunity to improve freedom, flexibility of dialysis, and well-being and strengthen family relationships [7]. These characteristics also may be beneficial to the PD group. In addition, ADL status is a predictive factor in choosing between dialysis and conservative treatment for CKD patients [37]. We believe that patients with independent ADL may be more likely to receive PD therapy because of the flexibility and well-being provided by this dialysis modality. In our study, we identified that the ADL rating was an independent factor altering the selection of RRM.

The timing of creating dialysis access might also influence the choice of RRM. Studies have shown that a considerable percentage (50%–60%) of ESRD patients who intended to choose PD ultimately received HD as their long-term treatment modality [18, 19]; of these patients, more than one-third initiated with emergent hemodialysis because of critical condition [19]. In addition, planned dialysis was associated with a higher proportion of PD and a higher percentage of vascular access preparation for HD compared with unplanned dialysis [9]. In our data, the HD patients had a high percentage of vascular access preparation before dialysis (compared with PD patients with peritoneal catheter implantation). We believe that the operation of vascular access is less suffering and it changes less body-image than PD catheter implantation for advanced CKD patients. Therefore, the optimal timing of dialysis access creation to predetermine ESRD patients remained a problem that might inhibit selection of another therapy modality. Conversely, the development of an embedded PD catheter procedure or emergent PD catheter implantation might increase the penetration rate of PD therapy.

Although all of the patients in the study received MPE, the percentage of using temporary dialysis access remained high (52.6%). Recent studies have recommended that MPE should provide sufficient information and be cautious against relying on dialysis patient experience sharing [38–40]. In addition, educators should focus on patient and family preparation, dialysis knowledge and the lifestyle implications of different RRM choices, and reconceptualization of the problems [40–42]. A study for dialysis patient in central Taiwan showed that the cognition of dialysis (the advantage and disadvantage of dialysis modality) and receptivity to dialysis (body-image change, personal privacy, the frequency of going to hospitals, the equipment of medical service in dialysis clinics, and the freedom of dialysis schedule) were the factors associated with RRM selection [10]. In our MPE program, educators provided adequate information and created a small group teaching session to help patient or family/caregiver to realize these topics. In our study, CKD patients who received small group teaching sessions and dialysis patients' experience sharing had an approximately 15% increase in dialysis preparation, but this was nonsignificant (*P* = 0.09). However, this data was limited by a small sample size (*n* = 36). We think that the reasons of CKD patients that make their final choices of RRM are multifactorial. Further work is necessary to determine the reasons for this phenomenon and to develop an effective education model.

Our analysis has several limitations. This was a single center study, which may limit generalizability to centers with different predialysis educational programs, access to facilities, areas of medical and nonmedical services, and policies on RRM selection. In addition, there was the possibility of bias in the data collection for this retrospective study. However, part of the data was collected prospectively and patient follow-ups were complete. We aimed to identify the factors influencing RRM selection using the patient's medical characteristics, but more information on the socioeconomic, psychological, and environmental factors that might alter the patient's preference was not included. This is the first study to estimate physician bias by using actual RRM selections by CKD patients and not a questionnaire. In addition, we described a new CKD education program and evaluated the efficacy of the program related to RRM selection and dialysis access preparation. Our study also carries important implications for using GIS to study the spatial distribution of dialysis patients.

## 5. Conclusions

This study indicates that the crucial patient characteristics influencing treatment of peritoneal dialysis included age, education level, and ADL level, but not the nephrologist recommendation or traveling time to clinics. The timing of a vascular access operation may also influence RRM selection. We recommend further research to explore the specific reasons for CKD patients changing their choices between the time of initial RRM selection and actual dialysis initiation.

## Figures and Tables

**Table 1:** The characteristics of the incident patients with end-stage renal disease.

	Patients number	Dialysis modality		*P* value
		HD (*n* = 524)	PD (*n* = 132)	
Age at the first dialysis (year)				

Mean ± SD	64.77 ± 12.63	66.32 ± 11.72	58.61 ± 14.18	<0.001
<50 yr	72 (11.0)	42 (8.0)	30 (22.7)	
50–59 yr	150 (22.9)	109 (20.8)	41 (31.1)	
60–69 yr	170 (25.9)	139 (26.5)	31 (23.5)	
≥70 yr	264 (40.2)	234 (44.7)	30 (22.8)	
Gender (%)				0.913
Male	360 (54.9)	287 (54.7)	73 (55.3)	
Female	237 (45.2)	237 (45.2)	59 (44.7)	
Employment (%)				<0.001
Retired or unemployed	543 (82.8)	449 (82.7)	94 (17.3)	
Yes	113 (17.2)	75 (14.3)	38 (28.8)	
Education (%)				<0.001
Noneducation	238 (36.3)	207 (39.5)	31 (23.5)	
Primary school & junior high school	324 (49.4)	260 (49.6)	64 (48.5)	
Senior high school & college	94 (14.3)	57 (10.9)	37 (28.0)	
ADL (%)				0.011
Independent	544 (82.9)	423 (80.7)	121 (91.7)	
Partial dependent	96 (14.6)	87 (16.6)	9 (6.8)	
Dependent	16 (2.4)	14 (2.7)	2 (1.5)	
Marital status (%)				0.546
Married	586 (89.3)	470 (89.7)	116 (87.9)	
Unmarried	70 (10.7)	54 (10.3)	16 (12.1)	
Body mass index (BMI, kg/m^2^)	24.49 ± 3.82	24.56 ± 3.97	24.2 ± 3.20	0.342
BMI status (%)				0.149
<24.00	225 (47.1)	173 (45.4)	52 (53.6)	
≥24.00	253 (52.9)	208 (54.6)	45 (46.4)	
Comorbidity disease (%)				0.076
=0	82 (12.5)	63 (12.0)	19 (14.4)	
=1	199 (30.3)	150 (28.6)	49 (37.1)	
≥2	375 (57.2)	311 (59.4)	64 (48.5)	
Traveling time^a^ (%)				0.703
<16 min	253 (38.6)	202 (38.5)	51 (38.6)	
16–30 min	238 (36.3)	189 (36.1)	49 (37.1)	
31–45 min	117 (17.8)	97 (18.5)	20 (15.2)	
>45 min	48 (7.3)	36 (6.9)	12 (9.1)	
Small group teaching session				0.108
No	620 (94.5)	499 (80.5)	121 (19.5)	
Yes	36 (5.5)	25 (69.4)	11 (30.6)	
Dialysis access preparation				<0.001
Nonpreestablishment	345 (52.6)	249 (47.5)	96 (72.7)	
Preestablishment	311 (47.4)	275 (52.5)^b^	36 (27.3)^c^	

^a^Estimated by Geographic Information System (GIS).

^b^Predialysis AV fistula or AV graft operation.

^c^Predialysis peritoneal catheter implantation.

**Table 2:** Multivariate logistic regression analysis for odds of PD.

	Odds ratio	95% CI	*P* value
Age at the first dialysis (year)			
<50 yr	3.309	(1.683–6.507)	0.001
50–59 yr	2.214	(1.244–3.940)	0.007
60–69 yr	1.409	(0.794–2.502)	0.241
≥70 yr (reference group)	1.000		
Education			
Senior high school & college	2.454	(1.297–4.642)	0.006
Primary school & junior high school	1.104	(0.663–1.838)	0.703
Noneducation (reference group)	1.000		
ADL			
Partially dependent	0.438	(0.205–0.934)	0.033
Dependent	0.899	(0.188–4.310)	0.894
Independent (reference group)	1.000		

**Table 3:** The dialysis patients follow-up by different nephrologist.

	Patients number	Dialysis modality		*P* value
		HD (*n* = 524)	PD (*n* = 132)	
Nephrologist (%)				0.256
1	101	84 (83.2)	17 (16.8)	
2	120	89 (74.2)	31 (25.8)	
3	84	72 (85.7)	12 (14.3)	
4	138	112 (81.2)	26 (18.8)	
5	213	167 (78.4)	46 (21.6)	
